# Pathological Mechanisms of Particulate Matter-Mediated Ocular Disorders: A Review

**DOI:** 10.3390/ijms252212107

**Published:** 2024-11-11

**Authors:** Jung-Hwa Han, Chaima Amri, Hyesook Lee, Jin Hur

**Affiliations:** 1 Department of Convergence Medicine, Pusan National University School of Medicine, Busan 50612, Republic of Korea; 2 PNU GRAND Convergence Medical Science Education Research Center, Pusan National University School of Medicine, Yangsan 50612, Republic of Korea; 3 Research Institute for Convergence of Biomedical Science and Technology, Pusan National University Yangsan Hospital, Yangsan 50612, Republic of Korea

**Keywords:** air pollution, fine dust, oxidative stress, ocular surface, retinal disorders, particulate matter

## Abstract

Air pollution presents a severe risk to public health, with particulate matter (PM) identified as a significant hazardous element. However, despite the eye organ being constantly exposed to air pollution, only recently has the impact of PM on ocular health caught the attention of researchers and healthcare professionals. By compiling pertinent data, this paper aims to enhance our understanding of the underlying pathological mechanisms of PM-mediated ocular disorders and facilitate the development of effective treatment strategies. Recent data support the association between exposure to PM and the development of ocular pathologies such as dry eye syndrome, retinal atherosclerosis, and glaucoma. Based on the results of multiple studies, PM exposure can lead to oxidative stress, inflammation, autophagy, cell death, and, ultimately, the development of ophthalmic diseases. This review aims to consolidate the latest findings on PM-mediated ocular diseases by summarizing the outcomes from epidemiological, in vitro, and in vivo studies on ocular surface and retinal disorders as well as other relevant ophthalmic disorders.

## 1. Introduction

With the development of modern society, air pollutant particulate matter (PM) has been recognized as a major environmental problem posing a serious threat to human health and quality of life [1,2]. PM refers to inhalable particles, composed of sulphate, nitrates, ammonia, sodium chloride, black carbon, mineral dust, or water [3]. PM is generally defined by its aerodynamic diameter, with PM_2.5_ and PM_10_ being the most common in the regulatory framework and relevant for health. The number after PM refers to the aerodynamic diameter of the particles; i.e., PM_10_ refers to particles <10 μm, and PM_2.5_ refers to particles less than 2.5 μm [4,5,6]. Sources of the largest particles, called coarse particles (particles with a diameter between 2.5 µm and 10 µm), mainly consist of pollen, sea spray, and wind-blown dust from erosion, agricultural spaces, roadways, and mining operations. The finer particles (i.e., PM_2.5_) can be derived from combustion sources such as wood and biomass fuels [3,4]. In 2013, following a study conducted by the International Agency for Research on Cancer, the World Health Organization established new guidelines, where PM, a major component of outdoor air pollution, was classified as a group 1 carcinogen [7]. PM_2.5_ enters the bronchi, passes the alveoli, and easily moves to other organs in the body through the blood circulation, leading to greater toxicity than fine dust [8]. According to many epidemiological and toxicological studies, exposure to PM_2.5_ increases not only early mortality [9,10] and cancer risks [11] but also the prevalence of skin [12], respiratory system [13], nervous system [14], vascular [15], and cardiovascular diseases [16].

The eye, being constantly exposed to the external environment, is particularly susceptible to the effects of air pollution. According to some epidemiological studies, exposure to severe air pollution, even for short periods of time, can cause symptoms such as itching, irritation, and foreign body sensation in the eyes [4,17]. Other studies reported a close relationship between air pollution and an increase in the number of ophthalmology outpatients [18,19]. Notably, the PM composition induces various pathological conditions in the eyes [20]. This review outlines the current research trends on ocular diseases resulting from exposure to fine dust. By compiling pertinent data, this paper aims to enhance our understanding of the underlying pathological mechanisms and facilitate the development of effective treatment strategies.

## 2. Methodology

**Figure 1:**
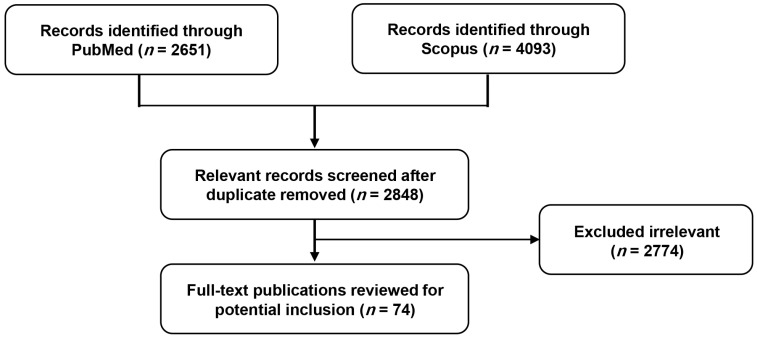
Flow chart of the literature selection process.

We conducted a comprehensive literature search across databases such as PubMed and Scopus. Key search terms included “particulate matter” or “air pollution”, in combination with terms such as “eye”, “ocular”, “dry eye syndrome”, “conjunctivitis”, “retina”, “glaucoma”, “cataract”, and “pterygium”. We included all relevant studies published from 2000 to September 2024, spanning experimental, clinical, and epidemiological studies (Figure 1). Studies related to indoor PM and smoking-related health issues were excluded from this overview. The search results were restricted to publications in English. In this review, we focused on the effects of outdoor PM on ocular disorders and analyzed the underlying mechanisms from both epidemiological and toxicological perspectives.

## 3. Particulate Matter-Mediated Ocular Diseases

**Figure 2:**
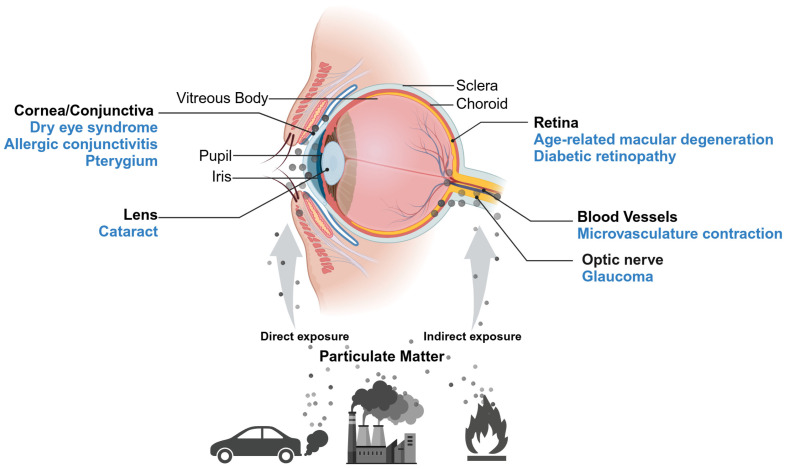
Anatomy of the eyeball and a schematic representation of the effects of particulate matter on ocular diseases.

PM enter all parts of the eye through direct exposure of the ocular surface or indirect exposure via inhalation, ultimately leading to various ocular disorders. In this review, we summarized the outcomes from epidemiological, in vitro, and in vivo studies on PM-mediated ocular diseases (Figure 2).

### 3.1. Ocular Surface Disease Caused by PM

The ocular surface, composed of the cornea, conjunctiva, corneoscleral limbus, and tear film, is in contact with the external environment and plays a role in various anatomical, physiological, and immunological protective functions [21]. Trauma, infection, and numerous environmental factors, can cause irritation resulting in corneal and conjunctival dysfunction characterized by symptoms such as pain, itching, foreign body sensation, decreased vision, or blindness [22]. An accumulation of epidemiological studies have reported an increased ocular surface disease index; dry eye syndrome symptoms such as foreign body sensation, stinging, redness, and itching; and exacerbated allergic keratoconjunctivitis cases in people exposed to fine dust [4,17,18,19,23,24].

#### 3.1.1. PM-Mediated Dry Eye Disease (DED)

DED is a multifactorial ocular surface disease where an inflammatory response is caused by a disrupted osmotic pressure and overall homeostasis of the tear film [21,25]. In people exposed to PM_2.5_, noticeable changes in the Schirmer test values, tear volume, and tear break-up time indicating an impairment in the tear film, consequently affecting the ocular surface, have been reported, but not in PM_10_-exposed subjects [22] (Table 1). A prospective cohort study with 43 DED patient in Korea suggested that each air pollutant (PM_2.5_ and PM_10_) may aggravate DED via different mechanisms of action [4,17,18,19,23,24,26]. Their findings revealed that increased PM_10_ exposure was only associated with decreased TBUT, but there was no effect on tear secretion and the corneal fluorescein staining score (CFSS). However, increased PM_2.5_ exposure was positively correlated with the OSDI score and led to aggravated ocular discomfort. Similarly, in a prospective, multiple cohort study with 387 DED participants in China, the ocular surface disease index (OSDI) score and tear secretion were positively correlated with PM_2.5_ exposure, and exposure resulted in ocular discomfort and damage with tear film instability [24]. They also demonstrated that PM_2.5_ and PM_10_ exposure led to aggravated meibomian gland dysfunction and upregulated tear cytokine levels. More recently, a retrospective cohort study with 53 DED patients showed that exposure to higher PM_2.5_ levels was associated with higher symptom assessment in DED (SANDE) and tear osmolarity [27].

Accumulating experimental studies showed that there is a direct correlation between PM exposure and DED. Recent studies confirmed that short-term topical exposure to 100 μg/mL PM_2.5_ is toxic to corneal epithelial cells (CECs) [28] (Table 2). Additionally, the administration of PM_2.5_ for 6 months to C57BL/6 mice causes ocular surface changes similar to DED [28]. The tear film consists of a lipid layer produced by the meibomian glands, an aqueous layer secreted by the lacrimal glands, and a mucin layer from the goblet cells of the conjunctiva [29]. An in vivo study reported that following the topical administration of 5 mg/mL PM_10_ and 100 μg/mL PM_2.5_ in mice, a disruption of the tear film accompanied by more severe repercussions, notably a delayed healing of the corneal epithelial layer and significant damage to the lacrimal glands causing inflammatory reactions, were observed [30,31]. More recently, Lee et al. [32] demonstrated that topically administered 5 mg/mL diesel PM_2.5_ promotes tear film destabilization, a reduction in the conjunctival goblet cells, and inflammation of the lacrimal gland in Sprague–Dawley rats. In Tu et al.’s work, C57BL/6J mice treated with urban PM eye drops suffered from ductal occlusion, glandular dropout, and lipogenic disorder, which are all indicators of meibomian gland dysfunction [33]. Tau et al. [34] reported that exposure to diesel fine dust at concentrations of 10–500 μg/mL caused cytotoxicity in corneal epithelial cells. The same study reported an increased expression of inflammatory cytokines and decreased expression of mucins (MUC1 and MUC16), indicating that tear film instability was most likely caused by a decrease in the mucus content leading to an increase in the ocular surface area exposed to fine dust damaging the corneal epithelial layer. Taken together, these studies provide a clearer understanding of the multifaceted impact of fine dust on each of the tear film components disrupted in DED.

#### 3.1.2. PM-Mediated Conjunctivitis

Allergic conjunctivitis, a typical inflammatory ocular surface disease, is caused by a reaction of the ocular immune cells to various allergens such as pollen, airborne dust, mold, food, and spores [35,36]. Clinical symptoms include itching, redness, burning sensation, and swelling of the conjunctiva and eyelids. According to several epidemiological studies, when in contact with the ocular surface, fine dust exceeding 10 μg/m^3^ in the air can potentially cause acute and chronic conjunctivitis, increasing the morbidity of patients suffering from such allergy [18,19] (Table 1). A global review article by Alryalat et al. reveals that PM_10_ was the pollutant most widely agreed upon to be associated with a higher frequency of conjunctivitis [37]. In their review, multiple studies of PM_2.5_ found a significant association with higher conjunctivitis-related clinic visits. Nevertheless, a single study found no association, possibly related to the technique used for PM_2.5_ quantification, which is dependent on meteorological conditions.

In a study on the process by which PM could trigger allergic reactions, Tang et al. [36] reported that following 18 days of exposure to PM_2.5_ (3.2–12.8 mg/mL), ICR mice developed eyelid edema, ocular surface damage, goblet cell hyperproliferation in the upper palpebral conjunctiva only, and extensive eosinophil infiltration in the entire conjunctiva and meibomian glands. A more recent study reported that PM could exacerbate the severity of allergic reactions in ovalbumin-sensitized C57BL/6 mice by causing eyelid edema and the upregulation of interleukin (IL)-4, IL-5, Tumor necrosis factor alpha (TNF-α), and serum immunoglobulin E [38]. Interestingly, this animal model demonstrated not only a diminished number of goblet cells in the conjunctiva but also greater mast cell degranulation and polymorphonuclear leukocyte (PMN) infiltration [38]. Furthermore, both the conjunctiva and cornea tissue of the mice exhibited a significantly higher number of apoptotic cells and uncontrolled cell proliferation of the basal epithelial layer. This same phenotype has been previously observed in the immortalized human conjunctival epithelial cell line IOBA-NHC exposed to diesel fine dust (10–500 μg/mL) [34] (Table 2). The upregulation of inflammatory markers such as intercellular adhesion molecule-1 (ICAM-1), IL-6, and IL-8 has been reported in both immortalized and primary human conjunctival epithelial cells [34,39].

#### 3.1.3. PM-Mediated Pterygium

Pterygium is an eye disease characterized by a benign growth of fibrous vascular tissue from the conjunctiva toward the center of the cornea [40]. Although the exact pathogenesis of pterygium is not yet known, ROS induced by ultraviolet-B, low humidity, and dust are known causes of tissue chronic inflammation and neovascularization [40,41]. In 2017, Lee et al. [42] investigated the correlation between air pollution and pterygium in 23,276 adults from 2008 to 2011 and reported that increased exposure to fine dust was linked to the prevalence of pterygium, although this study observed no significant association between air pollution and overall pterygium or pterygium recurrence in Korean adults (Table 1). Meanwhile, a case–control study reported by Ramirez et al. [5] in 2018 did not observe a correlation. However, this study had limitations, as it did not quantify the total inhalable fine dust and was based on the diagnosis established by an attending physician rather than an ophthalmologist. In 2021, Fu et al. [43] reported that significant associations between outpatient visits for pterygium and PM (PM_2.5_ and PM_10_) were observed. Furthermore, they demonstrated that younger patients were found to be more sensitive to air pollution, especially female patients during the warm season. Based on their finding, gender- and season-specific effects were observed between certain air pollutants and outpatient visits for primary pterygium, indicating a possible relationship between the incidence of primary pterygium and air pollution.

### 3.2. Particulate Matter-Mediated Retinal Disorders

The retina is an innervated membrane that shares many similarities with the brain. Its role is to convert light energy into electrical signals and transmit them to the brain through the optic nerve. In this neural tissue, nutrients and oxygen are received through the choroid, while metabolites are discharged through the retinal pigment epithelium [44]. The retina consists of 10 layers: an inner limiting membrane (ILM), a nerve fiber layer (NFL), a ganglion cell layer (GCL), an inner plexiform layer (IPL), an inner nuclear layer (INL), an outer plexiform layer (OPL), an outer nuclear layer (ONL), an outer limiting membrane (OLM), a photoreceptor layer (PL), a retinal epithelial layer, and a retinal pigmented epithelium (RPE) [44].

#### 3.2.1. Experimental Studies of Particulate Matter-Mediated Retinal Disorders

The RPE constitutes the outer wall of the blood–retinal barrier (BRB) and plays a role in phagocytosis, the production of growth factors, and the transport of oxygen and nutrients to the photoreceptor cells in the upper layer [45]. Damage to the RPE can cause degradation of the retinal nerves and photoreceptors, ultimately leading to retinal dysfunction and vision loss [46]. Gu et al. [47,48] investigated the impact of airborne fine particulate matter on retinal health in two separate studies reporting the damage caused to both the inner and outer BRB. The team was the first to report that PM_2.5_ exposure increased retinal vascular permeability and the diameter of the inner BRB of mouse models while decreasing the viability, proliferation, migration, and angiogenesis and increasing apoptosis and inflammation in human retinal microvascular endothelial cells (HRMECs) in a dose-dependent manner. Moreover, PM_2.5_ exposure induced iron overload and lipid oxidation, altering prostaglandin-endoperoxide synthase 2, glutathione peroxidase 4, ferritin heavy chain 1, and ferroptosis-related gene expression [47] (Table 3). In their second study, the team reported that mice treated with PM_2.5_ eye drops are more at risk of retinal edema due to an increased choroidal vasodilation and tight junction impairment. Noteworthy, continuous exposure caused gradual RPE repair but induced symptoms akin to age-related macular degeneration (AMD) [48]. In another animal model, Sprague–Dawley rats, acute respiratory exposure to 200 mg/mL diesel PM for 1 h increased the thickness of the OPL, INL, and ONL, causing hypoxia-mediated retinal edema [49]. Following 6 months of exposure to concentrated ambient PM_2.5_, C57BL/6 mice exhibited a poor response to light stimuli, retinal thinning, and a reduction in the expression of the retinal ganglion cell-selective marker RBPMS [50]. In addition, Lee et al. [32] demonstrated that the population of ganglion cells, as well as the thickness of the NFL, GCL, and IPL, markedly decreased by exposure to topically administered 5 mg/mL diesel PM_2.5_ in Sprague–Dawley rats (Table 4). To investigate its effects on human retinal development, an hESC-derived retinal organoid was exposed to varying doses of PM_2.5_. This fine dust exposure negatively influenced cell proliferation and apoptosis, resulting in reduced organoid size and neural retina thickness in a dose-dependent manner and causing dislocation of retinal ganglion cells and overall structural disorder [51]. Similarly, zebrafish embryos exposed to PM exhibited morphological defects, such as reduced body length and eye area, due to the dysregulation of embryogenesis and developmental gene expression [52].

#### 3.2.2. Epidemiological Studies of Particulate Matter-Mediated Retinal Disorders

Although the relationship between fine dust and disease morbidity has not been clearly evaluated, multiple cohort studies in highly polluted areas have established a correlation between air pollution and the risk of developing AMD [53,54,55]. In 2020, Pan et al. [56] published the results of a study on the effect of fine dust exposure on diabetic retinopathy, a vascular disease characterized by damage to the blood vessels of the retina (Table 1). Their case–control study followed the disease progression of 5790 newly diagnosis diabetic patients over a year post-diagnosis and established a correlation between the average exposure to fine dust and the risk of developing diabetic retinopathy. A small cohort study including 683 participants (75 years or older) who were assessed over a period of 11 years showed that higher PM_2.5_ and black carbon exposure correlated with faster NFL thinning, further confirming the link between air pollution and retinal neurodegeneration [57]. Meanwhile, a cohort study on 51,710 adults (40–69 years old) assessing changes in retinal structure according to air pollutant (PM_2.5_, PM_2.5–10_, PM_10_, and NO_2_) exposure reported thickening of the retinal NFL and a thinning of the GCL, IPL, INL, OPL, and ONL in people exposed to higher concentrations of PM_2.5_ fine dust [58]. Interestingly, the retinal vessel diameter is considered an independent risk factor for cardiovascular disease, as the retinal vessels share anatomical and physiological characteristics with coronary vessels and can provide insights into overall cardiovascular health [59,60]. Several epidemiological studies have shown that long-term exposure to 3 μg/m^3^ PM_2.5_ for 2 years narrows the retinal artery diameter and causes retinal atherosclerosis, while short-term exposure to PM_2.5_ (3 μg/m^3^) and PM_10_ (10 μg/m^3^) causes retinal arteriosclerosis, suggesting that retinal microvascular abnormalities are related to cardiovascular disease [61,62]. In 2017, 221 school-age children took part in a study on chronic exposure to fine particulate air pollution, and the results demonstrated that an increase of 10 μg/m^3^ in daily PM_2.5_ concentration correlated with a 0.35 μm decrease in the retinal microvasculature diameter [63]. A recent study reported a significant correlation between central retinal artery occlusion (CRAO) and the concentrations of NO_2_ and PM_2.5_ and lower ambient air temperature. However, this study relied mainly on retrospective data, and further experimental work is necessary to confirm such an association [64]. Recent research has established that PM_2.5_ particles can pass through the placenta of pregnant women, potentially leading to premature birth, low birth weight, and, in some cases, stillbirth [65]. Particulate matter has been found to impact the retinal structure during the early development stages in cellular and animal models.

### 3.3. Particulate Matter-Associated Glaucoma

Glaucoma is a condition characterized by visual field defects resulting from optic nerve dysfunction. Although elevated intraocular pressure can impair the blood flow to the optic nerve, the precise cause remains unidentified [66]. Wang et al. [67] identified a correlation between PM_2.5_ and glaucoma, while Min et al. [68] reported that both short-term and long-term exposure to PM_10_ can cause pediatric glaucoma, and Wu et al. established that there are significant associations between long-term exposure to PM_2.5_, PM_10_, and SO_2_ and increased acute primary angle-closure risk [69] (Table 1). A cross-sectional study on 33,701 adults in rural China reported that a 10 μg/m^3^ increase in PM_2.5_ was associated with a 7% higher risk of glaucoma and a 14% higher risk of primary angle-closure glaucoma [70]. Interestingly, the results of an epidemiological study targeting 111,370 UK Biobank participants, indicated that exposure to an average of 10 μg/m^3^ PM_2.5_ could cause glaucoma through neurotoxicity and vascular dysfunction caused by mechanisms unrelated to an increased intraocular pressure [71]. More recently, a Taiwan-based study demonstrated that increased PM_2.5_ exposure was related to the development of primary open angle glaucoma risk [72].

In vitro, human trabecular meshwork (HTM) cell contraction and cell viability are decreased when exposed to PM_2.5_, while C57BL/6 mouse topically treated with PM_2.5_ presented an increased intraocular pressure and NOD-like receptor protein 3 (NLRP3) inflammasome, caspase-1, IL-1β, gasdermin D (GSDMD), and ROS levels [73]. While the link between particulate matter (PM) and glaucoma has been established, research on the correlation with fine dust remains limited, and the underlying mechanisms still need clarification.

### 3.4. Particulate Matter-Associated Cataract

Cataract, another disorder affecting the vison, occurs when the eye’s lens clouds over, typically resulting from genetic predisposition, aging, trauma, or systemic diseases [74]. In an attempt to investigate the relationship between the cataract morbidity rate and atmospheric pollution (PM_10_, O_3_, NO_2_, and SO_2_), 18,622 Koreans over 40 years of age were enrolled. The morbidity rate increased concomitantly with O_3_ but not with the fine dust concentration [75].

**Table 1:** Summary of the outcomes from epidemiological studies on PM-mediated ocular disorder.

Subjects	Exposure Condition		Outcome	Major Findings	Ref.
	Materials	Dose and Time			
5062 DED outpatients form China (Hangzhou)	PM_2.5_ PM_10_	Continuous daily urban exposure	DED	Higher PM exposure linked to increased DED visits, especially in cold season	[17]
78 volunteers from Argentina	PM_2.5_ PM_10_	17.1 ± 8.4 μg/m^3^ for a minimum of 14 h daily 41.4 ± 16.4 μg/m^3^ for a minimum of 14 h daily	DED	↑ Bulbar redness by PM_2.5_ ↑ Eyelid redness by PM_2.5_ ↑ Degree of vital staining with fluorescein (SF) and lissamine green (SLG) by PM_2.5_	[22]
43 DED patients from Korea	PM_2.5_ PM_10_	1 μg/m^3^ for 1 day, 1 week, or 1 month	DED	↑ Ocular surface disease index (OSDI) score and ocular discomfort by PM_2.5_ ↓ Tear film break-up time (TBUT) and tear film stability by PM_10_	[23]
387 DED patients from China	PM_2.5_ PM_10_	1 μg/m^3^ for 1 day, 1 week, or 1 month	DED	↑ Schirmer’s I test (ST) by PM_2.5_ ↓ Tear meniscus height (TMH), TBUT, and meibomian gland (MG) function by PM_2.5_ and PM_10_ ↑ Tear cytokine levels by PM_2.5_ and PM_10_	[24]
53 DED patients from Korea	PM_2.5_	17.2 ± 7.7 μg/m^3^ for daily life	DED	↑ SANDE score ↑ Tear osmolarity No changes in tear secretion and TBUT	[27]
9737 conjunctivitis outpatients form China (Hangzhou)	PM_2.5_ PM_10_	Continuous daily urban exposure	Conjunctivitis	↑ Conjunctivitis outpatient visits by increased PM_2.5_ and PM_10_ levels Higher PM exposure linked to various impacts; stronger in cold season and in ages 2–5	[18]
77,439 emergency department (ED) visits for conjunctivitis in Canada	PM_2.5_	Continuous daily urban exposure	Conjunctivitis	↑ PM_2.5_ linked to higher ED visits for conjunctivitis	[19]
23,276 adults in Korea	Ambient PM_10_	Continuous daily exposure	Pterygium	↑ Exposure to higher PM10 levels was associated with primary pterygium	[42]
3017 outpatients with pterygium in China (Hangzhou)	PM_2.5_ PM_10_	Continuous daily urban exposure	Pterygium	↑ Significant associations between outpatient visits for pterygium and PM Younger patients were found to be more sensitive to PM2.5 exposure, especially females during the warm season	[43]
4,284,128 adults aged 50–80 in Taiwan	PM_2.5_	Annual mean PM_2.5_ exposure of 34.23 ± 7.17 μg/m^3^ over 11 years	AMD	10 μg/m^3^ increase in PM_2.5_ led to a 19% higher AMD risk	[54]
15,115 adults aged 40+ in Korea	PM_10_	Annual mean PM_10_ exposure of 49.52 µg/m^3^ over 5 years	AMD	↑ PM_10_ exposure was borderline significant with early AMD prevalence Higher PM_10_ levels may increase AMD risk	[55]
Diabetic patients in Taiwan	PM_2.5_ PM_10_	Average concentration of PM_2.5_ (28.9–38.0 μg/m^3^) and PM_2.5–10_ (21.7–29.4 μg/m^3^) over 5.6 years	DR	10 μg/m^3^ increase in PM_2.5_ and PM_2.5–10_ was linked to 1.29 and 1.37 times higher DR risk, respectively	[56]
631 elderly adults (average age 82) in France	PM_2.5_	10-year average exposure of PM_2.5_ (median: 21.9 μg/m^3^)	Neurodegeneration	Higher PM_2.5_ levels were linked to faster retinal nerve fiber layer (RNFL) thinning (−0.28 μm/year)	[57]
51,710 adults aged 40–69 in UK	PM_2.5_ PM_10_	Annual average concentration of PM_2.5_ (median 9.92 µg/m^3^) and PM_10_ (median 19.72 µg/m^3^), µg/m^3^	Neurodegeneration	Higher PM_2.5_ levels were associated with thicker RNFL and thinner ganglion cell inner plexiform layer (GCIPL) and inner nuclear layer (INL)	[58]
4607 adults aged 46–87 in U.S.	PM_2.5_	Long-term average PM_2.5_ concentration over 2 years	Narrower retinal arteriolar diameter (CRAE) and wider venular diameter (CRVE)	Long-term PM_2.5_ exposure was linked to a 0.8 µm decrease in CRAE	[61]
84 healthy adults aged 22–63 from Belgium	PM_10_	Short-term PM_10_ exposure in 24 h	Changes in retinal arteriolar (CRAE) and venular (CRVE) diameters	10 µg/m^3^ increase in PM_10_ was linked to a 0.93 µm decrease in CRAE and a 0.86 µm decrease in CRVE	[62]
221 school-aged children, aged 8–12 in Belgium	PM_2.5_	Same-day exposure measured at school (average 16.8 μg/m^3^) and chronic exposure modelled at residence (annual mean 15.4 μg/m^3^)	Changes in retinal arteriolar (CRAE) and venular (CRVE) diameters	10 μg/m^3^ increase in same-day PM_2.5_ exposure decreased CRAE by 0.35 μm and increased CRVE by 0.35 μm Chronic PM_2.5_ exposure showed a trend toward wider venules	[63]
432 patients with central retinal artery occlusion (CRAO) in Germany	PM_2.5_	PM_2.5_ concentrations analyzed over 15 years, with higher exposure in winter months	CRAO	Higher PM_2.5_ concentrations were linked to increased CRAO cases, peaking in winter, especially in February	[64]
Pregnant women	PM_2.5_	PM_2.5_ exposure during pregnancy	Increased risks of low birth weight (LBW), preterm birth (PTB), and stillbirth	↑ PM_2.5_ exposure was linked to a 9% increase in LBW risk for each 10 μg/m^3^ increment PTB risk increased, especially during the third trimester, due to oxidative stress and inflammation Higher PM_2.5_ levels correlated with an increased risk of stillbirth	[65]
9004 infants in Korea (Seoul)	PM_10_	Annual average: 65.1 μg/m^3^ and 54.7 μg/m^3^ 11-year follow-up study	Glaucoma	Glaucoma occurred in 85 patients (0.94%) Increases of 1 μg/m^3^ of long-term PM10 were significantly associated with increased hazard ratios (HRs) for childhood glaucoma	[68]
281 patients with acute primary angle closure (APAC) in China	PM_2.5_ PM_10_	Annual average of PM_2.5_ and PM_10_: 37.25 µg/m^3^ and 51.91 µg/m^3^	Glaucoma	Long-term exposure to ambient air pollutants increased the risk of APAC	[69]
33,701 adults in China	PM_2.5_	Annual average: 62 μg/m^3^	Glaucoma	Increased odds of glaucoma and primary angle-closure glaucoma (PAGG) were associated with high PM_2.5_ pollution	[70]
1320 patients diagnosed with primary open-angle glaucoma (POAG) in Taiwan	PM_2.5_	Normal level: <25 μg/m^3^/month WHO level 1: ≥1 to <1.5 × 25 μg/m^3^/month WHO level 1.5: ≥1 to <1.5 × 25 μg/m^3^/month WHO level 2: ≥ 2 × 25 μg/m^3^/month	Glaucoma	As the PM2.5 level rises, POAG risk increases, and it is significant at the WHO 2.0 level	[72]

↑: Increased; ↓: decreased.

**Table 2:** Summary of the outcomes from in vitro studies on PM-mediated ocular surface disorder.

Cells	Exposure Condition			Major Findings	Ref.
	Materials	Concentration	Exposure Time		
Human corneal epithelial cells (Human CECs)	PM < 4 μm (SRM 2786)	0–200 μg/mL	12 h and 24 h	↑ Apoptosis ↑ Intracellular reactive oxygen species (ROS)	[28]
Human CECs	Collected PM < 2.5 μm	0–20–50–100 μg/mL	0–24 h	↓ Cellular mobility ↓ Actin reorganization formation via ROS ↓ Interaction of cytoskeleton proteins	[30]
Human CECs	Collected atmospheric PM_2.5_	500 μg/mL	0–48 h	↓ Proliferation ↑ Latex bead-positive phagocytic cells	[31]
Human CECs	Collected diesel exhaust particles (DEPs)	0–500 μg/mL	24 h	↓ Viability and proliferation ↑ Pro-inflammatory cytokine IL-6 ↓ MUC1 and MUC16	[34]
Human CECs	Collected PM_2.5_	0–800 μg/mL	0–48 h	↓ Proliferation ↑ Mitochondrial ROS (mtROS) ↓ Mitochondrial membrane potential (∆*Ψm*) and ATP production ↑ Inflammation ↑ Nrf2 degradation and NF-κB activation	[76]
Human CECs	Collected atmospheric PM_2.5_	0–100 μg/mL	0–48 h	↓ Cell viability and proliferation ↑ Apoptosis ↓ Autophagy in the early stage ↑ Autophagy in the late stage	[77]
Human CECs	Collected atmospheric PM_2.5_	0–100 μg/mL	0–48 h	↑ Autophagy via overexpression of plasminogen activator inhibitor type-2 (PAI-2) ↑ Inflammatory genes ↑ Aryl-hydrocarbon stimulatory genes	[78]
Human CECs and primary bovine CECs	Collected PM < 2.5 μm	20, 50, 100 and 200 μg/mL	24 h	↑ Cytotoxicity ↑ DNA damage (DNA double-stand breaks and DNA repair-related protein γH2AX) ↑ Intracellular ROS ↑ Cell senescence	[79]
Primary human CECs	Collected indoor dust < 100 μm	5–320 μg/100 μL	24 h	↑ Cytotoxicity ↑ Intracellular ROS ↑ Oxidative stress markers (malondialdehyde and 8-hydroxy-2-deoxyguanosine) ↓ Antioxidant capacity ↑ Inflammatory mediators (IL-1β, IL-6, IL-8, TNF-α, and MCP-1) ↑ Oxidative DNA damage ↑ Mitochondrial dysfunction	[80]
Primary rat CECs	Diesel PM_2.5_ (SRM1650b)	0–300 μg/100 μL	24 h	↑ Cytotoxicity ↑ Inflammatory mediators ↑ Intracellular ROS ↑ Oxidative DNA damage ↑ Mitochondrial dysfunction ↑ p38MAPK/NF-κB signal activation via ROS	[81]
Human conjunctival epithelial cells	Diesel exhaust particles	100 μg/mL	24 h	↑ Inflammatory mediators (intercellular adhesion molecule 1, IL- 6, and IL-8)	[39]

↑: Increased; ↓: decreased.

**Table 3:** Summary of the outcomes from in vitro studies on PM-mediated retinal disorder.

Cells	Exposure Condition			Major Findings	Ref.
	PM	Concentration	Exposure Time		
Human retinal pigment epithelial ARPE-19 cells	Diesel PM_2.5_ (SRM1650b)	0–50 μg/mL	24 h	↑ Morphology alteration ↑ Cell migration ↓ Epithelium markers ↑ Mesenchymal markers ↑TGF-β/Smad/ERK/p38 MAPK signaling pathway ↑Epithelial–mesenchymal transition (EMT) via ROS-dependent mechanism	[82]
ARPE-19 cells	PM_10_-like (ERM-CZ120)	0–500 μg/mL and 200 mg/mL	0–8 h	↑ Inflammatory mediators ↑Endoplasmic reticulum (ER) stress markers ↑Endoplasmic reticulum stress markers ↑Cytosolic [Ca^2+^]_i_ level ↑ Phosphorylation of MAPK/NF-κB axis via ER-related unfolded protein response (UPR) pathways	[83]
AREP-19 cells	Urban aerosols (CRM28)	0–200 μg/mL	24 h	↑ Cytotoxicity ↑ Autophagy and mitophagy ↑ Necrosis ↑ G2/M phase cell cycle arrest ↑ DNA and mitochondrial damage ↑ ROS-mediated cellular dysfunction	[84]
AREP-19 cells	Urban aerosols (CRM28)	0–300 μg/mL	48 h	↑ Cellular senescence ↑ Intracellular ROS ↑ mtROS	[85]
Human retinal microvascular endothelial cells	Collected ambient PM_2.5_	0–100 μg/mL	24 and 36 h	↑ Cytotoxicity ↑ Inflammasome formation ↓ Migration and angiogenesis ↑ Ferroptosis ↑ Iron overload and lipid peroxidation	[47]
Human embryonic stem cell-derived retinal organoids (hEROs)	Urban PM_2.5_ (SRM 1648a)	0–100 μg/mL	3 weeks	↓ Differentiation of hERO-derived neural retina ↓ Areas of hEROs ↓ Ki67-positive proliferative cells ↑ TUNEL-positive apoptotic cells ↑ Structural disorder of hERO-derived neural retina ↑ MAPK and PI3K/Akt signal activation ↓ Fibroblast growth factors (FGF8 and 10)	[51]

↑: Increased; ↓: decreased.

**Table 4:** Summary of the pathological mechanisms of PM-mediated ocular disorders in vivo.

Animals	Exposure Condition			Major Findings	Ref.
	Materials	Concentration	Exposure Time		
Sprague–Dawley rats (Male, 8 weeks old)	Collected ambient PM_2.5_	1 mg/mL PM_2.5_, 10 μL per eye	4 times/day for 3 days	↓ Vascular permeability via dysfunction of the inner blood–retinal barrier ↑ Retinal inflammation	[47]
Sprague–Dawley rats (Female, 8 weeks old)	Collected atmospheric PM_2.5_ samples	10 μL of 1 mg/mL PM_2.5_	Topical eye drops, 4 times/day for 21 days	↓ Tear secretion ↑ Corneal surface damage ↑ PAI-2 ↑ Autophagy-related markers	[78]
Sprague–Dawley rats (Female, 6 weeks old)	Diesel PM_2.5_ (SRM1650b)	20 μL of 5 mg/mL PM_2.5_	Topical eye drops, 4 times/day for 14 days	↓ Tear secretion ↑ Corneal surface damages ↓ Conjunctival goblet cell population ↑ Inflammation of lacrimal gland ↑ Retinal ganglion cell loss ↓ Thickness of NFL/GCL/IPL in the retina	[32]
BALB/c mice (Male, 18–21 g body weight)	Collected atmospheric PM_2.5_	5 mg/mL	Topical eye drops, 4 times/day for 14 days	↓ Tear secretion ↑ Corneal surface damages ↑ Inflammatory index in ocular surface ↑ Epithelium edema and apoptosis in the cornea and conjunctiva ↓ Conjunctival goblet cell population ↑ Apoptosis of lacrimal glands	[31]
C57BL/6J mice (Female, 6–8 weeks old)	PM < 4 μm (SRM 2786)	0.5, 1.0, and 5.0 mg/mL	Topical eye drops, 4 times/day for 6 months	↓ Tear secretion and tear break-up time ↑ Corneal surface damage ↓ Conjunctival goblet cell population ↑ inflammatory cytokines in corneal and conjunctival tissue	[28]
C57BL/6J mice (Female, 6–8 weeks old)	Urban PM_2.5_ (SRM1648a)	12 mg/mL	Topical eye drops, 4 times/day for 14 days	↑ Meibomian gland (MG) dysfunction ↑ Corneal surface damage ↑ Apoptosis of MG cells ↑ Hyperkeratinization and ductal blockage ↑ Neutrophil recruitment to surrounding microenvironment of MG ↑ NLRP3-mediated pyroptosis via p38 MAPK/NF-κB signaling pathway	[33]
C57BL/6J mice (Male, 6 weeks old)	Collected PM_2.5_	N/A	PM free/PM room exposure 8 h/day for 3, 7, and 10 weeks	↑ Corneal damage ↑ Corneal epithelium detachment ↑ Corneal inflammation ↑ NF-κB activation in cornea	[76]
C57BL/6 wild type mice (Male, 8–12 weeks old)	Collected PM < 2.5 μm	100 μg/mL	Topical administration every 4 h after corneal abrasion for 64 h	↓ Cornea wound healing	[30]
C57BL/6J mice (Female, 8 weeks old)	PM < 4 μm (SRM 2786)	3.0 mg/mL	Topical eye drops, 3 times/day for 7 days	↑ Eyelid edema ↑ Mast cell degranulation ↑ Inflammatory cytokines and serum IgE ↑ Apoptosis and reduced goblet cells	[38]
ICR mice (Female, 6–8 weeks old)	Collected PM_2.5_	3.2, 6.4, and 12.8 mg/mL	Topical eye drops, 3 times/day for 19 days	↑ Clinical scoring of allergic conjunctivitis ↑ Conjunctival goblet cell population ↑ Eosinophil infiltration	[36]

↑: Increased; ↓: decreased.

## 4. Pathological Mechanisms of Ocular Damage Caused by PM

**Figure 3:**
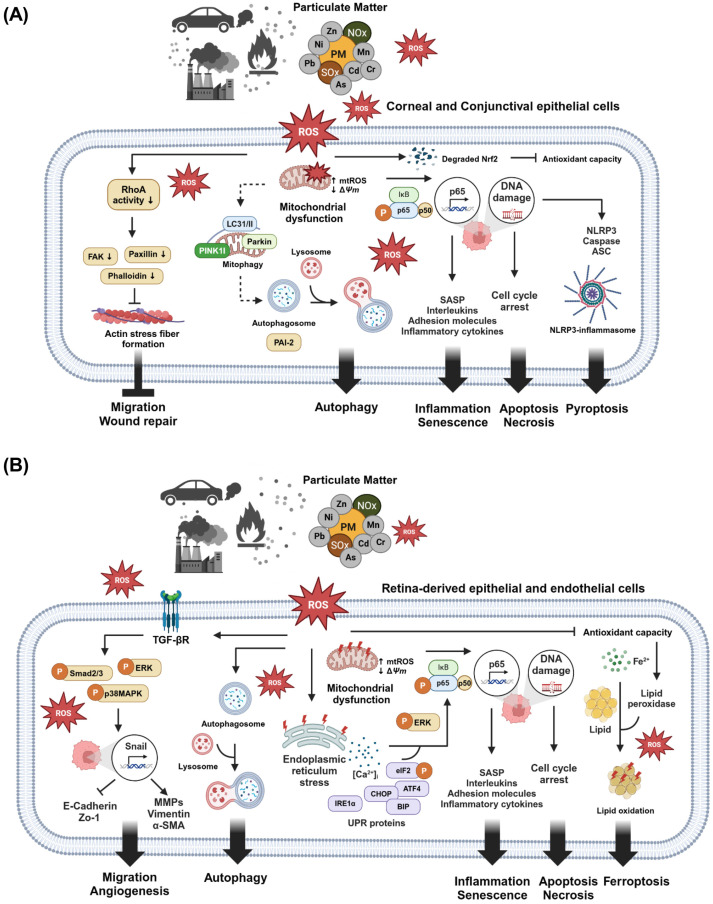
Understanding the pathological mechanisms of particulate matter-mediated ocular disorders. (**A**) Illustration of how PM-induced ROS regulate cellular pathological events, such as autophagy, inflammation, senescence, cell death, and pyroptosis, on the ocular surface. (**B**) Summary of how PM-induced ROS regulate cellular pathological events in the retina.

In this review, we describe the representative mechanisms of PM that induce the cellular pathological events on the ocular surface and retina based on key findings from in vitro studies (Figure 3, Table 2 and Table 3).

### 4.1. Oxidative Stress

Reactive oxygen species (ROS) are known to cause damage to macromolecules such as lipids, proteins, RNA, and DNA [86,87]. ROS can be generated in mitochondria or on the surface of metal particles. The heterocyclic polycyclic aromatic hydrocarbons (PAHs), nitro-PAHs, and various metals (iron, copper, chromium, and vanadium, etc.) contained in fine dust are known to react together generating ROS. More precisely, PAHs and nitro-PAHS are adsorbed on the surface of the metal particles catalyzing Fenton and Fenton-like reactions. ROS generated by metals such as the ones found in fine dust have been shown to damage the eye tissue [87,88]. Somayajulu et al. [89] showed that ROS generated from human CECs treated with PM_2.5_ upregulated the expression of antioxidant enzymes (heme oxygenase-1, catalase, superoxide dismutase, and glutathione S-transferase, etc.) to activate the protective mechanisms against oxidative damage. In contrast, Xiang et al. [80] showed that house dust, including fine dust, exhibited ROS-induced cytotoxicity in CECs not only causing malondialdehyde (MDA) and 8-hydroxy-2-deoxyguanosine (8-OHdG) upregulation but also decreasing antioxidative capacity by inhibiting the production of antioxidant enzymes and decreasing mitochondrial membrane potential (∆*Ψm*) (Table 2). According to a study by Yang et al. [28], PM_2.5_ treatment in CECs significantly increased the production of ROS while inducing apoptosis both in a dose- and time-dependent manner. Furthermore, Lee et al. established that ROS generated by PM_2.5_ in human retinal epithelium ARPE-19 cells caused mitochondrial damage and contributed to retinal dysfunction through the promotion of the transforming growth factor-β (TGF-β)/Smad/extracellular signal-activated kinase (ERK)/p38 mitogen-activated protein kinase (MAPK) signaling pathway, leading to epithelial–mesenchymal transition (EMT) [82] (Table 3). EMT is one of the major mechanisms causing RPE dysfunction and is involved in various fibrotic disorders of the eye, such as age-related macular degeneration, diabetic retinopathy, and proliferative vitreoretinopathy [90]. Multiple studies reported that exposure to fine particulate matter induces EMT whether in pulmonary epithelial cells or cancer cells, enhancing pathological responses through cellular invasion and mobility [91,92]. To date, Lee et al. [82] are the only ones to investigate the pathological mechanism of oxidative stress-mediated EMT in retinal damage caused by fine particulate matter. In human CECs, PM_2.5_ triggered dose- and time-dependent proliferation inhibition, ROS generation, and mitochondrial dysfunction, leading to inflammation cascade activation. Nuclear factor erythroid 2-related factor 2 (Nrf2) overexpression and Nuclear factor kappa B (NF-κB) P65 knockdown mitigated inflammation by regulating ROS levels and downstream inflammatory pathways, highlighting potential therapeutic targets for PM_2.5_-induced DED [76]. Another study suggested that ARPE-19 exposure to urban particulate matter (UPM) promoted cellular senescence mediated by mitochondrial reactive oxygen species-dependent activation of the NF-κB pathway [85]. The study reported increased senescence-associated β-galactosidase activity and the upregulation of senescence markers (p16 and p21) and the senescence-associated secretory phenotype including IL-1β and matrix metalloproteinase (MMP)-1 and MMP-3 [85]. Furthermore, Kim et al. [81] demonstrated that diesel PM_2.5_ promotes inflammation and cellular dysfunction through the ROS/p38 MAPK pathway in primary rat CECs. Cui et al. [30] reported that PM_2.5_-induced mouse ocular surface damage inhibits cell mobility by blocking the FAK/paxillin/Rho A signaling pathway in human CECs, ultimately delaying wound healing. Additionally, it was established that ROS played a major role in inhibiting the wound healing mechanism. Another study confirmed that, in CECs, DNA double helix breaks, γH2AX expression, and cell senescence were noticeably increased following exposure to PM_2.5_, and recovery was possible when treated with N-acetyl-l-cysteine, a known potential ROS scavenger [79].

### 4.2. Inflammation

Ocular inflammation caused by particulate matter (PM) exposure can lead to various symptoms and diseases. Understanding the mechanistic pathways behind such pathologies is a necessary step in developing possible treatments. Multiple studies have reported a broad spectrum of ocular inflammatory responses induced by PM exposure. At the cellular level, in murine models, fine dust has been consistently reported to cause inflammatory cell infiltration and activation of the MAPK/NF-κB signaling pathway regulating the expression of various cytokines and adhesion molecules involved in inflammatory responses [31,33,65,83,93]. The corneal tissues of PM_2.5_- and PM_10_-treated mice have shown not only clinical symptoms similar to dry eye syndrome but also an upregulated expression of TNFα and NF-κB p65, along with an increase in inflammatory cell infiltration [31,65]. Tau et al. confirmed an increased expression of IL-6 and a decreased expression of MUC1 and MUC16 in CECs exposed to fine dust [34], while Fujishima et al. [39] reported the upregulation of IL-6 and ICAM-1. However, neither of the authors identified a clear mechanism of action. The research results of Yang et al. [28] also showed that the production of IL-18, IL-22, IL-23, and monocyte chemoattractant protein-1 (MCP-1) significantly increased on the ocular surface of mice treated locally with PM. In human retinal epithelium ARPE-19 cells, PM exposure activates the MAPK/NF-κB axis and increases TNFα, IL-1β, IL-6, and monocyte chemoattractant protein 1 (MCP-1) mRNA expression, indicating elevated inflammation. PM also induces ER stress, as indicated by the increased [Ca^2+^]_i_ levels and upregulation of the unfolded protein response (UPR) pathways. These findings suggest that PM exposure promotes ocular inflammation and stress adaptation in retinal cells, providing insights into the molecular mechanisms of PM-induced ocular pathologies [83]. Due to the complexity of the inflammatory pathways triggered by fine dust, identifying a single pathway is challenging. Nevertheless, we have gained valuable insights into the molecular mechanisms of PM-induced inflammation, highlighting key players involved in the process.

### 4.3. Autophagy

Through autophagy, the cell removes any cellular waste, unnecessary proteins, or dysfunctional cell organelles. This process plays a critical role in development, aging, cell homeostasis, and various physiological and pathological mechanisms [94]. In the eye organ, autophagy is known to be involved in decomposing invading microorganisms while maintaining corneal homeostasis and transparency [95,96]. The involvement of autophagy induced by fine dust has been linked to cardiovascular and respiratory diseases, and it has been suggested that autophagy inhibitors could alleviate symptoms caused by PM_2.5_ [97,98,99]. Through their work, Fu et al. [77] showed that in CECs exposed to PM_2.5_, despite a slight downregulation during the early stages, the autophagy-related markers microtubule-associated protein light chain 3 (LC3) I/II, autophagy-related gene (ATG) 5, and Beclin 1 (BECN1) were significantly increased during later stages of exposure. Human CECs exposed to PM_2.5_ at a concentration above 100 μg/mL upregulated the production of ROS, causing DNA damage and cell aging [79], and at a concentration as low as 50 μg/mL, cytotoxicity was increased by inducing autophagy [77]. Lyu et al. [78], through RNA sequencing analysis, identified 434 differentially expressed genes in PM_2.5_-exposed human corneal epithelial cells. Among these genes, plasminogen activator inhibitor type-2 (PAI-2) expression was up-regulated and correlated with the autophagy markers LC3 II and BECN1, suggesting its role in PM_2.5_-induced autophagy [78].

### 4.4. Cell Death

Under normal conditions, programmed cell death is a fundamental mechanism ensuring tissue homeostasis. However, dysregulation triggered by various environmental stressors leading to excessive cell death can disrupt normal physiological processes. Recent studies have highlighted the role of PM in inducing cell death across ocular tissues, emphasizing different mechanisms such as apoptosis and ferroptosis. On the ocular surface, PM exposure exacerbates inflammatory responses, disrupting tear film and cell integrity, which promotes apoptosis. In a mouse model of allergic eye disease, exposure to PM significantly increased the number of apoptotic cells in the conjunctiva and cornea tissue [38]. It has also been reported that PM mediates CEC apoptosis both in a dose- and time-dependent manner [28]. In human embryonic stem cell-derived retinal organoids, PM_2.5_ exposure reduced neural retina formation and thickness due to increased apoptosis and decreased cell proliferation [51]. In contrast, PM seems to impact human RPE cells differently. A study on ARPE-19 cells found that UPM exposure led to G2/M cell cycle arrest, DNA damage, and mitochondrial dysfunction-induced cytotoxicity. However, increased necrosis and autophagy but not apoptosis was reported [84]. Interestingly, in human retinal microvascular endothelial cells (HRMECs) exposed to PM_2.5_, an increase in Terminal deoxynucleotidyl transferase dUTP nick-end labeling-positive apoptotic cells was observed along with a significantly altered expression of ferroptosis-related genes, such as prostaglandin-endoperoxide synthase 2, glutathione peroxidase 4, and ferritin heavy chain 1, pointing to ferroptosis, a type of iron-dependent cell death distinct from apoptosis [47]. Overall, these findings clearly demonstrate that PM is associated with various forms of cell death across different ocular tissues. Despite these insights, research is still lacking and must focus on elucidating the specific context of each mechanism involved.

## 5. Discussion

The eye is one of the few organs constantly exposed to the external environment. Fine dust particles enter all parts of the eye through direct exposure of the ocular surface or indirect exposure via inhalation, ultimately leading to various ocular disorders. In this review, we summarized the outcomes from epidemiological, in vitro, and in vivo studies on PM-mediated ocular diseases. Furthermore, we provide the representative mechanisms of PM that induce ocular pathology based on the findings of previous in vitro and in vivo studies. Ophthalmic pathologies caused by fine dust are rooted in molecular mechanisms such as oxidative stress, inflammatory responses, autophagy, and cell death. Clearly, the multilayer effect of fine dust exposure adds to the complexity of studying it and eventually establishing therapeutic strategies for PM-induced ocular diseases. Nonetheless, the findings reported by epidemiological and clinical studies underscore the urgent need for targeted interventions and treatment development to mitigate environmental air pollution’s detrimental effects on ocular health.

In conclusion, while significant progress has been made in understanding the pathological mechanisms of PM on ocular disorders, much remains to be explored. The primary limitation of this review stems from the results of epidemiological studies. Experimental studies can more accurately control the type, concentration, and duration of PM exposure, leading to more consistent results. However, most epidemiological studies rely heavily on the types and concentrations of PM in the atmosphere, which are affected by climatic factors such as temperature, humidity, wind speed, and atmospheric pressure, as well as geographical location. Therefore, while some studies report statistically significant adverse effects of PM on eye health, others do not show this correlation, possibly due to insufficient sample sizes rather than a true lack of negative impact. Overcoming the limitations of previous studies through more advanced research methods and exploring potential therapeutic strategies will be essential in developing practical approaches to mitigate the harmful effects of PM on eye health.

## Data Availability

Data sharing not applicable—no new data generated.
